# Health of unpaid carers in Wales, UK: a population data linkage study

**DOI:** 10.1093/pubmed/fdad207

**Published:** 2023-11-02

**Authors:** Fangzhou Huang, Jiao Song, Alisha R Davies

**Affiliations:** School of Management, Swansea University, Swansea SA1 8EN, UK; The Communicable Disease Surveillance Centre, Public Health Wales, Cardiff CF10 4BZ, UK; Research and Evaluation Division, Knowledge Directorate, Public Health Wales, Cardiff CF10 4BZ, UK

**Keywords:** carers, morbidity and mortality, public health

## Abstract

**Background:**

The population of unpaid carers in Wales increased to record. There is no systematic approach to record unpaid caring status, resulting in limited quantitative evidence on unpaid carers’ health. The aim of this study is to: (i) create an e-cohort of unpaid carers by linking routinely collected health and administrative datasets in Wales, UK. (ii) investigate whether long-term health conditions and multimorbidity are more prevalent amongst unpaid carers than non-carers.

**Methods:**

Unpaid carers were identified by linking primary care dataset, National Survey for Wales data with demographic characteristics in the Secure Anonymise Information Linkage Databank. The clinical codes identified in Cambridge Multimorbidity Score were used to explore the prevalence of long-term health conditions.

**Results:**

A total of 91 220 unpaid carers in Wales were identified between 1 January 2010 and 1 March 2022. Unpaid carers were found at higher risk of managing 35 of 37 long-term health conditions and multimorbidity than non-carers, exacerbated amongst younger age groups and deprived communities.

**Conclusions:**

The creation of the first e-cohort of unpaid carers in Wales provides opportunities to perform rapid analysis to systematically understand health needs and evaluate initiatives in future. To better support unpaid carers, flexible approaches focusing on early identification and prevention is crucial.

## Introduction

With an ageing population and increasing proportion managing complex health needs, unpaid carers play a significant role in providing care. Unpaid carer is ‘a person looks after, or give any help or support to family members, friends, neighbours or others’.^1^ It was estimated that there were over 400 000 unpaid carers in 2019 in Wales, UK and approximately 700 000 during the COVID-19 pandemic with the number expected to keep increasing in a foreseeable future.^2^^,^^3^

Providing care can be rewarding whilst having negative impact on unpaid carers’ own health but often overlooked.^4^^,^^5^ Studies found that high caring intensity had negative impact on unpaid carers mental wellbeing, and unpaid carers were more likely to suffer from anxiety, depression and other physical health conditions than non-carers.^4^^,^^6–10^ However, there is limited quantitative studies investigating the health needs of unpaid carers. Particularly, there is a lack of systematic data collection on unpaid carers in Wales, therefore conducting large scale data analysis to understand the inequalities and unpaid carers’ health condition becomes extremely difficult.^11^

This study aims to generate a reproducible e-cohort of unpaid carers in Wales utilizing routinely collected health and administrative data, and understand the health needs of this population group.

## Methods

### E-cohort of unpaid carers

**Fig. 1:**
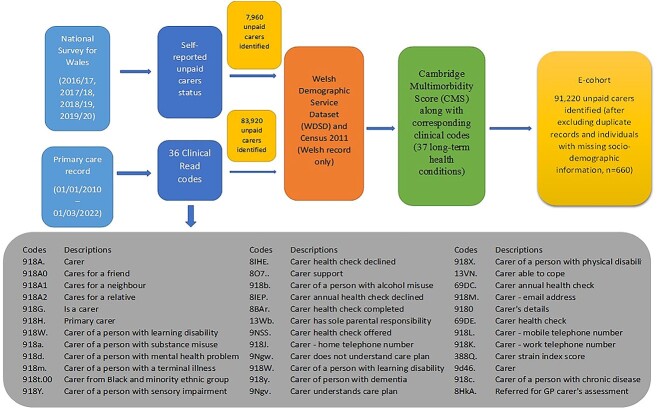
Flow diagram of the creation of the e-cohort of unpaid carers in Wales.

Working with general practitioners, a list of Read codes was validated to identify unpaid carers.^12^ National Survey for Wales (NSW) 2016/17, 2017/18, 2018/19 and 2019/20 data and primary care dataset (from 1 January 2010 to 1 March 2022) were used to identify unpaid carers (Fig. 1). Anonymous Linking Field code was utilized to eliminate duplication. Demographic Service Database and Census 2011 were linked to obtain socio-demographic information.

### A matched comparison group

Welsh residents who had not been identified as unpaid carers were randomly selected to form a comparison group matched with unpaid carers on sex, age and deprivation.^13^

### Long-term health conditions and multimorbidity

Clinical codes used to identify 37 long-term health conditions in Cambridge Multimorbidity Score were used to scan primary care records^14–16^ (full list of 37 long-term health conditions can be accessed from CPRD@Cambardge—Codes List^16^). Presenting with two or more conditions was defined as multimorbidity. We retrospectively tracked the primary care records of unpaid carers and flagged up cases with long-term health conditions and multimorbidity.

### Statistical analysis

In this study, all data were stored in the Secure Anonymised Information Linkage (SAIL) Databank where data linkage and analysis were conducted.^17^ Crude prevalence rate, adjusted prevalence rate, rate ratio of long-term health conditions and multimorbidity were produced. *P* < 0.05 was considered as statistically significant.

## Results

### E-cohort characteristics

Amongst 91 220 unpaid carers, approximately two-thirds were female (65.0% versus 50.7% in the general population) and a quarter were between 55 and 64 years old (24.7% versus 12.8%). In total, 5.5% of them were under the age of 25 and 16.5% were over 75. Distribution across deprivation quintiles was similar with mid-2018 Welsh population estimate.^18^

### Prevalence of long-term health conditions and multimorbidity

#### Overall prevalence of long-term health conditions

Overall, the prevalence of all 37 long-term health conditions was significantly higher amongst unpaid carers than non-carers, except for peripheral vascular disease (*P* = 0.14) and multiple sclerosis (*P* = 0.64). The five most common conditions amongst unpaid carers were anxiety and/or depression (age-sex standardized rate^18^: 243.9 per 1000 population), hypertension (89.2), hearing loss (79.4), chronic kidney disease (72.6) and asthma (60.2) (Table 1). The conditions amongst non-carers were consistent with unpaid carers but at a lower prevalence. Amongst the 10 most prevalent conditions, the greatest difference between unpaid carers and non-carers were cancer (rate ratio 2.5, 95% CI 2.4–2.7), anxiety and/or depression (1.7, 1.7–1.8), constipation (1.7, 1.6–1.8) and musculoskeletal disorders (1.5, 1.4–1.6), see Table 1.

**Table 1:** Top 10 prevalent (crude rate and adjusted rate of per 1000 population) of long-term health condition amongst unpaid carers (whole sample and by age groups) and rate ratio against non-carers

	Carer				Non-carer				Carer versus Non-carer			
Whole sample	Crude rate	Adjusted rate	CI low	CI high	Crude rate	Adjusted rate	CI low	CI high	Rate ratio	CI low	CI high	*P*-value (difference in crude rate)
Anxiety and/or depression	276.1	243.9	240.0	247.7	180.6	142.7	139.9	145.6	1.7	1.7	1.8	<0.001
Hypertension	129.7	89.2	87.5	90.8	108.4	72.1	70.7	73.5	1.2	1.2	1.3	<0.001
Hearing loss	91.4	79.4	77.0	81.9	67.1	52.7	50.9	54.5	1.5	1.4	1.6	<0.001
Chronic kidney disease	111.0	72.6	71.1	74.0	99.3	64.2	62.8	65.5	1.1	1.1	1.2	<0.001
Asthma	58.5	60.2	57.8	62.7	50.4	46.1	44.1	48.1	1.3	1.2	1.4	<0.001
Diabetes	74.9	55.4	53.9	56.8	67.5	47.2	45.9	48.4	1.2	1.1	1.2	<0.001
Musculoskeletal disorders	67.5	54.1	52.2	56.0	48.2	35.8	34.3	37.2	1.5	1.4	1.6	<0.001
Irritable bowel syndrome	60.5	49.5	47.8	51.3	41.2	32.7	31.2	34.1	1.5	1.4	1.6	<0.001
Constipation	57.0	41.1	39.6	42.6	36.8	23.8	22.9	24.7	1.7	1.6	1.8	<0.001
Cancer (diagnosis in last 5 years)	50.8	36.2	35.0	37.4	22.2	14.3	13.6	15.0	2.5	2.4	2.7	<0.001
**<25 years**												
Anxiety and/or depression	189.9	177.6	167.0	188.2	87.1	78.1	70.9	85.4	2.3	2.0	2.6	<0.001
Asthma	67.7	68.2	60.9	75.4	41.8	41.2	35.5	46.9	1.7	1.4	2.0	<0.001
Hearing loss	58.0	58.6	51.8	65.4	27.7	28.3	23.5	33.1	2.1	1.7	2.6	<0.001
Learning disability	46.3	53.5	46.7	60.3	5.3	5.8	3.6	8.1	9.2	6.1	14.5	<0.001
Musculoskeletal disorders	32.5	32.0	27.0	37.0	13.5	14.4	10.9	17.9	2.2	1.6	3.0	<0.001
Irritable bowel syndrome	33.5	28.6	24.2	33.0	21.4	19.2	15.5	22.9	1.5	1.2	1.9	<0.001
Epilepsy	23.4	25.7	20.9	30.4	5.7	5.5	3.4	7.6	4.6	3.0	7.4	<0.001
Schizophrenia or bipolar disorder	15.8	18.2	14.1	22.2	3.0	3.0	1.4	4.6	6.0	3.4	11.6	<0.001
Psychoactive substance misuse (not alcohol)	16.4	16.7	13.0	20.4	6.9	7.4	4.9	10.0	2.3	1.5	3.5	<0.001
Constipation	14.3	15.5	11.8	19.2	4.0	3.7	2.0	5.3	4.2	2.5	7.5	<0.001
**25–34 years**												
Anxiety and/or depression	286.4	271.1	260.5	281.7	155.5	138.9	130.9	146.8	2.0	1.8	2.1	<0.001
Irritable bowel syndrome	77.5	66.1	60.6	71.6	51.3	40.4	36.3	44.5	1.6	1.4	1.9	<0.001
Asthma	59.6	55.7	50.3	61.1	45.1	40.3	35.8	44.8	1.4	1.2	1.6	<0.001
Hearing loss	46.8	46.5	41.4	51.6	25.7	25.0	21.2	28.7	1.9	1.5	2.3	<0.001
Learning disability	31.0	40.8	35.5	46.1	3.8	3.8	2.3	5.3	10.8	7.0	17.1	<0.001
Musculoskeletal disorders	36.4	36.4	31.8	40.9	20.9	20.7	17.2	24.2	1.8	1.4	2.2	<0.001
Psychoactive substance misuse (not alcohol)	27.1	33.1	28.4	37.9	19.0	22.8	18.9	26.7	1.5	1.2	1.8	<0.001
Schizophrenia or bipolar disorder	24.1	27.9	23.6	32.2	8.6	8.2	6.0	10.4	3.4	2.5	4.7	<0.001
Epilepsy	20.1	23.2	19.3	27.1	7.8	7.5	5.4	9.6	3.1	2.2	4.3	<0.001
Alcohol problems	20.1	23.2	19.3	27.1	16.2	17.3	14.0	20.5	1.3	1.0	1.7	0.070
**35–44 years**												
Anxiety and/or depression	306.6	289.4	280.5	298.3	182.7	168.3	161.1	175.5	1.7	1.6	1.8	<0.001
Irritable bowel syndrome	82.8	73.0	68.1	77.8	46.7	40.3	36.7	43.9	1.8	1.6	2.0	<0.001
Asthma	65.0	61.8	57.1	66.5	50.7	48.7	44.4	52.9	1.3	1.1	1.4	<0.001
Hearing loss	50.1	51.5	47.0	56.0	29.7	29.4	26.0	32.8	1.8	1.5	2.0	<0.001
Hypertension	46.0	50.3	45.7	54.8	28.0	28.0	24.7	31.3	1.8	1.5	2.1	<0.001
Musculoskeletal disorders	49.8	48.2	44.0	52.4	28.3	26.4	23.3	29.5	1.8	1.6	2.1	<0.001
Diabetes	30.3	31.7	28.1	35.3	19.6	19.7	16.9	22.5	1.6	1.3	1.9	<0.001
Thyroid disorders	38.8	31.4	28.4	34.5	29.0	23.2	20.6	25.8	1.4	1.2	1.6	<0.001
Schizophrenia or bipolar disorder	25.8	29.8	26.2	33.4	10.5	11.0	8.8	13.1	2.7	2.1	3.5	<0.001
Alcohol problems	22.9	27.0	23.5	30.4	18.5	21.6	18.5	24.7	1.2	1.0	1.5	0.022
**45–54 years**												
Anxiety and/or depression	306.6	289.5	282.6	296.3	207.6	188.1	182.4	193.9	1.5	1.5	1.6	<0.001
Hypertension	114.1	120.0	114.8	125.1	86.6	88.9	84.5	93.4	1.3	1.3	1.4	<0.001
Irritable bowel syndrome	71.8	63.8	60.2	67.3	47.2	41.6	38.7	44.4	1.5	1.4	1.7	<0.001
Musculoskeletal disorders	65.2	61.4	57.8	65.0	43.0	40.9	37.9	43.8	1.5	1.4	1.7	<0.001
Hearing loss	58.4	59.7	56.0	63.4	38.5	37.8	34.9	40.7	1.6	1.4	1.8	<0.001
Asthma	60.8	58.1	54.6	61.7	53.2	50.8	47.4	54.1	1.1	1.0	1.3	0.002
Diabetes	48.9	53.5	49.8	57.1	46.7	49.6	46.1	53.0	1.1	1.0	1.2	0.335
Thyroid disorders	50.8	43.5	40.6	46.4	36.0	29.9	27.5	32.2	1.5	1.3	1.6	<0.001
Chronic kidney disease	36.4	34.3	31.6	37.1	35.3	33.5	30.8	36.2	1.0	0.9	1.2	0.609
Constipation	36.4	33.9	31.2	36.7	20.6	18.4	16.4	20.3	1.8	1.6	2.1	<0.001
**55–64 years**												
Anxiety and/or depression	264.5	251.4	245.5	257.3	195.1	179.9	174.8	185.0	1.4	1.3	1.5	<0.001
Hypertension	163.0	170.7	165.4	176.0	138.7	144.6	139.7	149.5	1.2	1.1	1.2	<0.001
Hearing loss	77.6	82.6	78.7	86.5	56.1	56.6	53.4	59.8	1.5	1.4	1.6	<0.001
Musculoskeletal disorders	76.1	72.7	69.2	76.2	56.5	53.7	50.7	56.7	1.4	1.3	1.5	<0.001
Diabetes	65.0	72.5	68.8	76.3	75.0	81.8	77.9	85.7	0.9	0.8	1.0	<0.001
Chronic kidney disease	66.1	63.5	60.2	66.8	64.9	61.5	58.3	64.8	1.0	1.0	1.1	0.594
Irritable bowel syndrome	59.3	53.1	50.1	56.0	42.1	37.0	34.6	39.5	1.4	1.3	1.6	<0.001
Asthma	49.5	47.5	44.6	50.4	52.5	49.0	46.2	51.9	1.0	0.9	1.1	0.151
Thyroid disorders	54.6	46.8	44.2	49.5	44.5	37.2	34.9	39.6	1.3	1.2	1.4	<0.001
Cancer (diagnosis in last 5 years)	41.4	44.3	41.4	47.3	24.1	22.9	20.9	24.9	1.9	1.7	2.2	<0.001
**65–74 years**												
Anxiety and/or depression	259.2	251.5	243.8	259.2	169.8	161.1	154.7	167.6	1.6	1.5	1.7	<0.001
Hypertension	206.6	210.0	202.5	217.4	178.1	179.5	172.5	186.5	1.2	1.1	1.2	<0.001
Chronic kidney disease	161.3	159.9	153.3	166.5	142.9	141.5	135.2	147.7	1.1	1.1	1.2	<0.001
Diabetes	141.9	149.6	143.1	156.2	116.2	119.9	113.9	125.8	1.2	1.2	1.3	<0.001
Hearing loss	118.0	122.4	116.3	128.4	93.4	95.1	89.8	100.5	1.3	1.2	1.4	<0.001
Cancer (diagnosis in last 5 years)	102.7	108.1	102.4	113.8	38.7	39.6	36.0	43.1	2.7	2.4	3.0	<0.001
COPD	81.0	83.9	78.8	89.0	61.9	62.5	58.1	66.9	1.3	1.2	1.5	<0.001
Musculoskeletal disorders	85.4	81.8	76.9	86.6	65.4	62.9	58.6	67.2	1.3	1.2	1.4	<0.001
Coronary heart disease	68.6	74.2	69.3	79.1	53.8	57.8	53.5	62.2	1.3	1.2	1.4	<0.001
Diverticular disease of intestine	73.4	71.7	67.1	76.3	61.4	60.2	55.9	64.5	1.2	1.1	1.3	<0.001
**75+ years**												
Chronic kidney disease	377.3	377.3	369.5	385.0	328.1	328.0	320.5	335.5	1.2	1.1	1.2	<0.001
Anxiety and/or depression	272.1	272.0	264.9	279.0	177.9	177.8	171.7	183.9	1.5	1.5	1.6	<0.001
Hypertension	199.5	199.5	193.1	205.9	179.2	179.2	173.1	185.3	1.1	1.1	1.2	<0.001
Hearing loss	194.3	194.3	188.0	200.6	158.4	158.5	152.6	164.3	1.2	1.2	1.3	<0.001
Dementia	185.0	184.9	178.7	191.1	54.7	54.7	51.0	58.3	3.4	3.1	3.7	<0.001
Constipation	155.6	155.6	149.8	161.3	106.5	106.5	101.5	111.4	1.5	1.4	1.6	<0.001
Diabetes	153.6	153.6	147.9	159.4	126.9	127.0	121.6	132.3	1.2	1.1	1.3	<0.001
Cancer (diagnosis in last 5 years)	122.5	122.5	117.3	127.8	49.0	49.1	45.6	52.5	2.5	2.3	2.7	<0.001
Atrial fibrillation	119.2	119.2	114.1	124.4	102.5	102.6	97.7	107.4	1.2	1.1	1.2	<0.001
Coronary heart disease	116.7	116.7	111.6	121.8	86.9	87.0	82.5	91.5	1.3	1.2	1.4	<0.001
**Most deprived quintile**												
Anxiety and/or depression	338.0	287.6	279.1	296.0	237.6	184.2	177.7	190.8	1.6	1.5	1.6	<0.001
Hypertension	131.7	88.1	84.5	91.6	107.4	70.9	67.8	74.0	1.2	1.2	1.3	<0.001
Hearing loss	85.4	74.1	69.2	79.1	63.2	48.7	45.0	52.4	1.5	1.4	1.7	<0.001
Asthma	70.6	71.0	65.5	76.4	63.8	55.0	50.6	59.5	1.3	1.1	1.4	0.010
Chronic kidney disease	96.5	62.1	59.1	65.1	88.1	56.2	53.4	58.9	1.1	1.0	1.2	0.007
Diabetes	87.1	60.3	57.2	63.4	80.3	53.9	51.1	56.7	1.1	1.0	1.2	0.020
Irritable bowel syndrome	68.2	54.7	50.8	58.5	44.3	33.2	30.4	36.1	1.6	1.5	1.8	<0.001
Musculoskeletal disorders	66.9	53.5	49.5	57.5	49.5	35.6	32.6	38.6	1.5	1.3	1.7	<0.001
Constipation	63.3	42.7	39.8	45.6	40.6	26.2	24.0	28.3	1.6	1.5	1.8	<0.001
Cancer (diagnosis in last 5 years)	50.5	34.1	31.7	36.5	20.9	13.3	11.8	14.8	2.6	2.2	3.0	<0.001
**Least deprived quintile**												
Anxiety and/or depression	234.3	209.3	200.3	218.3	145.5	111.7	105.7	117.7	1.9	1.7	2.0	<0.001
Hypertension	127.1	87.1	83.3	90.9	110.4	71.5	68.3	74.7	1.2	1.1	1.3	<0.001
Hearing loss	97.2	79.9	74.2	85.6	75.8	56.7	52.4	61.1	1.4	1.3	1.6	<0.001
Chronic kidney disease	112.5	68.7	65.3	72.0	102.3	61.0	58.2	63.7	1.1	1.0	1.2	0.002
Asthma	50.4	54.8	48.9	60.7	43.1	40.8	36.3	45.3	1.3	1.1	1.6	0.001
Irritable bowel syndrome	57.1	52.0	47.2	56.8	37.8	31.0	27.7	34.4	1.7	1.4	1.9	<0.001
Musculoskeletal disorders	62.3	48.9	44.4	53.4	48.2	35.3	32.1	38.6	1.4	1.2	1.6	<0.001
Diabetes	63.5	46.1	42.8	49.4	60.1	41.2	38.5	43.9	1.1	1.0	1.2	0.180
Constipation	53.3	39.3	35.4	43.1	38.3	23.8	21.6	26.0	1.7	1.4	1.9	<0.001
Cancer (diagnosis in last 5 years)	53.8	38.1	35.1	41.1	26.8	16.9	15.3	18.5	2.3	2.0	2.6	<0.001

#### By age groups and deprivation

Anxiety and/or depression were the most prevalent condition in all age groups amongst unpaid carers except 75+ years group, with higher prevalence than non-carers. The difference between unpaid carers and non-carers decreased with increasing age. Rate ratio for anxiety and/or depression between unpaid carers and non-carers was 2.3 (95% CI 2.0–2.6) for under 25 years old and reduced to 1.5 (1.5–1.6) for 45–54 and over 75 year olds (Table 1). The prevalence of 8 conditions (amongst the top 10) was higher for unpaid carers living in the most deprived areas than the least except for hearing loss and cancer, e.g. anxiety and/or depression (287.6 per 1000 population versus 209.3 per 1000 population) and hypertension (88.1 versus 87.1). The difference between unpaid carers and non-carers was marginally higher in the most deprived areas except for anxiety and/or depression (rate ratios, the most: the least deprived quintile 1.6: 1.9) (Table 1).

#### Multimorbidity

Prevalence of multimorbidity was higher in unpaid carers compared to non-carers at all ages and deprivation quintiles. Over half of unpaid carers between the age of 64 and 75 live with multimorbidity (533.3 per 1000 population versus 391.9 amongst non-carers). The difference in multimorbidity was greatest amongst the younger population and declined with increasing age. Amongst the most deprived communities, unpaid carers under 25 had 3.5 times (CI 2.6–4.6) the rate of multimorbidity compared to non-carers and the rate ratio reduced to 1.3 (CI 1.2–1.4) for over 75. Within least deprived communities, rate ratio was 6.1 (CI 3.7–10.6) for under 25 and reduced to 1.3 (CI 1.2–1.4) for over 75 age group.

## Conclusion/discussion

### Main finding of this study

This study created a unique and reproducible e-cohort of unpaid carers in Wales with 91 220 identified. Findings suggested that unpaid carers experience poorer health comparing to non-carers and the gap exacerbated amongst younger groups and in deprived communities. The health needs of unpaid carers are usually overlooked due to the focus on the health of the carees and juggling with other responsibilities.

### What is already know to this topic

Previous studies found providing care have detrimental impact on unpaid carers’ own health and wellbeing, whilst with limited quantitative evidence.

### What this study adds

The e-cohort can be used for rapid analysis in research and evaluation towards tailor-made support for unpaid carers in future. From policy making perspective, health services and policy should take flexible approaches for unpaid carers to access healthcare and focus on early identification and prevention for highlighted groups and conditions.

### Limitations of this study

There are certain limitations with this study. The identification of unpaid carers in routine data relies on the caring status to be recognized and recorded in surveys and clinical records. There are systematic differences in recording non-health patient information across healthcare settings. These barriers contribute to underestimating population size and bias in quantifying health needs, but highlighting the vital need of standardized approaches in recognizing and recording unpaid caring status.

## Data Availability

All data involved in this study are from Secure Anonymised Information Linkage Databank as stated in Statistical Analysis section and Ethics Statement.

## Notes

**Fangzhou Huang**, Senior Lecturer

**Jiao Song**, Senior Scientist (Statistics)

**Alisha R. Davies**, Professor, Consultant in Public Health, Head of Research and Development
